# Disease-causing cystathionine β-synthase linker mutations impair allosteric regulation

**DOI:** 10.1016/j.jbc.2023.105449

**Published:** 2023-11-08

**Authors:** Joseph V. Roman, Romila Mascarenhas, Karanfil Ceric, David P. Ballou, Ruma Banerjee

**Affiliations:** Department of Biological Chemistry, University of Michigan Medical Center, Ann Arbor, Michigan, USA

**Keywords:** hydrogen sulfide, cystathionine-β-synthase, allostery, S-adenosylmethionine, heme, transsulfuration pathway, homocysteine, pyridoxal phosphate

## Abstract

Cystathionine β-synthase (CBS) catalyzes the committing step in the transsulfuration pathway, which is important for clearing homocysteine and furnishing cysteine. The transsulfuration pathway also generates H_2_S, a signaling molecule. CBS is a modular protein with a heme and pyridoxal phosphate-binding catalytic core, which is separated by a linker region from the C-terminal regulatory domain that binds S-adenosylmethionine (AdoMet), an allosteric activator. Recent cryo-EM structures reveal that CBS exists in a fibrillar form and undergoes a dramatic architectural rearrangement between the basal and AdoMet-bound states. CBS is the single most common locus of mutations associated with homocystinuria, and, in this study, we have characterized three clinical variants (K384E/N and M391I), which reside in the linker region. The native fibrillar form is destabilized in the variants, and differences in their limited proteolytic fingerprints also reveal conformational alterations. The crystal structure of the truncated K384N variant, lacking the regulatory domain, reveals that the overall fold of the catalytic core is unperturbed. M391I CBS exhibits a modest (1.4-fold) decrease while the K384E/N variants exhibit a significant (∼8-fold) decrease in basal activity, which is either unresponsive to or inhibited by AdoMet. Pre-steady state kinetic analyses reveal that the K384E/N substitutions exhibit pleiotropic effects and that the differences between them are expressed in the second half reaction, that is, homocysteine binding and reaction with the aminoacrylate intermediate. Together, these studies point to an important role for the linker in stabilizing the higher-order oligomeric structure of CBS and enabling AdoMet-dependent regulation.

The reverse transsulfuration pathway is integral to mammalian sulfur metabolism and comprises two pyridoxal 5′-phosphate (PLP)-dependent enzymes: cystathionine-β-synthase (CBS) and cystathionine γ-lyase (CTH). The pathway is believed to divert excess sulfur from the methionine cycle toward cysteine synthesis (Fig. 1*A*). CBS catalyzes the first step in this pathway by condensing homocysteine and serine, generating cystathionine (1). CTH then cleaves cystathionine to cysteine, α-ketobutyrate and ammonia. Both enzymes are promiscuous, exhibiting lax substrate and reaction specificities, and catalyze multiple reactions leading to H_2_S synthesis (2). Furthermore, both enzymes catalyze the β-elimination of cystine forming cysteine persulfide (3), while CTH is additionally capable of forming homocysteine persulfide from homocystine (4). As the committing enzyme in the transsulfuration pathway, CBS is a major regulatory hub; it is activated by S-adenosylmethionine (AdoMet) (5) and glutathionylation (6), but inhibited by sumoylation (7, 8), carbon monooxide (9, 10), nitric oxide (11) and nitrite (12).

**Figure 1:**
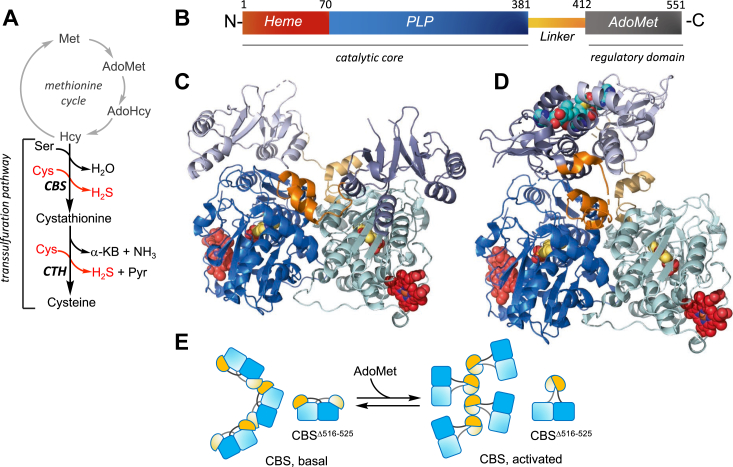
**Structures of dimeric human CBS in the basal and activated conformations.***A*, scheme showing the intersection between the methionine cycle (*grey*) and the transsulfuration pathway (*black*). For clarity, only the dominant H_2_S-generating reactions catalyzed by CBS and CTH are shown in *red*. α-KB is α-ketobutyrate. *B*, modular organization of CBS. *C* and *D*, the basal (PDB:4COO, *C*) and activated (PDB:4PCU, *D*) conformations of the CBS^Δ516–525^ dimer are shown using the same color scheme as in *B*. The two subunits are shown in dark and light shades. Heme, PLP, and AdoMet (in the activated conformation) are shown in *red*, *yellow*, and *cyan spheres*, respectively. *E*, schematic representation of the large conformational changes seen in the crystal structure of the CBS^Δ516–525^ dimer and the cryo-EM structure of the native fibrillar form. AdoMet either triggers or stabilizes the activated conformation in which the regulatory domains dimerize atop the catalytic cores, dramatically changing the organization of the fibrillar form.

CBS is unique among PLP enzymes in that it also harbors a heme cofactor (13) that is tethered *via* Cys-52 and His-65 coordination in the human protein, and held ∼20 Å from the active site PLP (14). The enzyme is organized into a catalytic core, which includes the heme and PLP cofactors (9, 15), and a regulatory domain with a tandem repeat of the Bateman (or CBS domain) module (16), which binds the allosteric activator, AdoMet (Fig. 1*B*). A 32-residue long interconnecting linker extends from amino acids 381 to 412 between the two domains.

Hereditary mutations in CBS lead to homocystinuria, an autosomal recessive disorder (OMIM 236200) that affects four major organ systems: CNS, ocular, vascular and skeletal (17). A large number of missense mutations map to the conserved catalytic core of CBS (18). Biochemical characterization of patient mutations has identified residues that contribute significantly to activity and/or regulation and provided insights into the mechanism underlying the PLP-responsive clinical phenotype (19, 20, 21). Several C-terminal regulatory domain variants retain robust catalytic activity that is equal to, or higher than that of wild-type CBS, but are insensitive to further activation by AdoMet (22, 23).

Crystal structures of CBS have revealed two major conformations, referred to as basal and activated (Fig. 1, *C* and *D*) (14, 24, 25, 26). Full-length human CBS is prone to aggregation and exists as a mixture of oligomeric states, ranging from 2- to ∼16-mers, which has been challenging for crystallographic studies. Deletion of a β-turn β-extension element spanning residues 516 to 525 in the regulatory domain, was key to crystallizing CBS in the basal conformation, while the additional introduction of the E201S mutation, led to the crystallization of the activated state with AdoMet bound (14, 24). In the basal state, the C-terminal regulatory domain from each subunit interacts with the catalytic core of the other and impedes active site access (Fig. 1*C*). In the activated state, the regulatory domains dimerize above the catalytic cores, enhancing active site access, while the connecting linkers provide the primary contacts between the N- and the C-terminal domains (Fig. 1*D*).

Full-length CBS, in the presence and absence of AdoMet, exhibits remarkable filamentous forms as revealed by cryo-EM analysis (27). In the basal state, a left-handed helical filament is observed in which the previously seen domain-swapped dimer represents the repeating unit (Fig. 1*E*). The 516 to 525 loop in each regulatory domain is engaged in oligomerization with the neighboring dimer. AdoMet elicits a significant change in filament morphology such that the regulatory domains form the central stalk while the dimeric catalytic domains from each unit protrude into solution (Fig. 1*E*). The relatively small magnitude of the AdoMet effect on CBS activity (2-fold) however, seems to be out of proportion with the apparently large gain in accessibility as suggested by the structures of the dimer or the full-length filament in the presence of AdoMet.

Previous studies have shown that deletion of the C-terminal regulatory domain or suppressor mutations in it alleviate the catalytic penalties associated with a number of common disease-causing mutations that localize to the catalytic core (28). These data suggest that impaired protein dynamics and the ability to sample alternate conformational and/or oligomerization states are compromised in some disease-causing CBS variants, and point to a potentially important role for the intervening linker sequence. However, unlike mutations in the catalytic and regulatory domains, the penalties imposed by mutations in the flexible linker are largely unknown. In this study, we have characterized a pair of missense mutations that localize at Lys-384 (K384E/N) as well as M391I, all of which reside in the interconnecting linker region. We find that the variants show a decreased propensity for forming higher-order oligomers, exhibit diminished catalytic activity, and surprisingly, are either unresponsive to AdoMet or inhibited by it.

## Results

### The linker mutations affect CBS oligomerization

**Figure 2:**
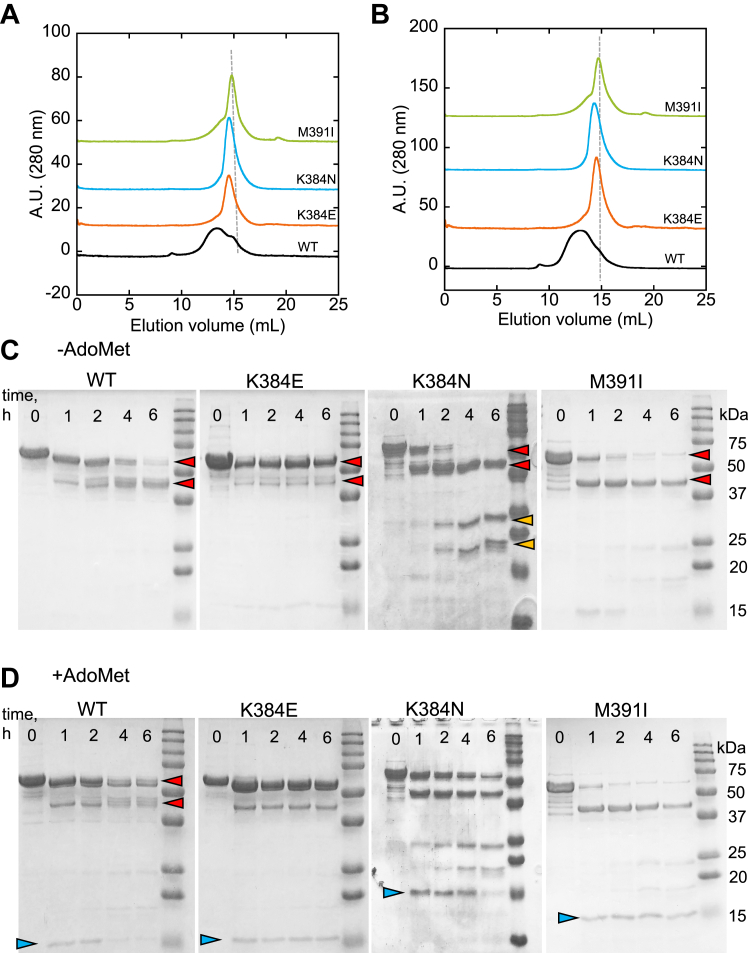
**The linker mutations affect CBS oligomerization and proteolytic sensitivity.***A*, wild-type CBS elutes primarily as higher-order oligomers while the K384E/N and M391I variants elute as narrower peaks. *B*, concentration dependence of the oligomeric distribution of wild-type *versus* K384E/N and M391I CBS. The proteins (80 μg in *A* and 240 μg in *B*) were separated on a Superose 6 Gl10/300 size exclusion column eluted with 50 mM Tris pH 8.0, 0.5 mM TCEP. The *dashed grey lines* in *A* and *B* denote the elution position for a peak with a molecular mass of ∼320 kDa. *C* and *D*, comparison of major bands produced after 1, 2, 4, and 6 h of limited proteolysis by trypsin of wild-type (WT) full-length CBS and linker mutants in the absence (*C*) and presence (*D*) of 380 μM AdoMet. The *red* (∼58 and ∼42 kDa), *yellow* (24 and 19 kDa) and *blue* (15 kDa) *arrowheads highlight* proteolytic fragments of the indicated sizes.

The yields of the purified K384E/N variants were similar to that of wild-type CBS (∼7.0 mg/liter of culture), while the M391I variant was obtained in lower yield (3 mg/l of culture). The purity of each variant was judged to be >90% based on SDS-PAGE analysis and a 428:280 nm (heme:protein) ratio of ∼1.0. In comparison to wild-type CBS, which elutes as a broad peak from a size exclusion column, consistent with the presence of a mixture of oligomeric states, K384E/N and M391I CBS eluted as narrower peaks, with the M391I variant centered at ∼320 kDa (Fig. 2*A*). The elution profile of wild-type CBS but not of the variants, was sensitive to protein concentration, and shifted further towards higher-order oligomers when the concentration was tripled (Fig. 2*B*).

Limited proteolysis can be a useful tool to probe for conformational changes that impact accessibility to hypersensitive sites. Initial cleavage of the 63 kDa wild-type CBS protomer occurs at Lys-30,-36, and −39 (29), leading to a cluster of bands centered at ∼58 kDa (Fig. 2*C*), which are further cleaved at Lys-413, *i.e.* at the boundary of the linker and regulatory domains (Fig. 2*C*, red arrowheads) (29). The resulting ∼42 kDa bands, represent the catalytic core and are stable up to 6 h. The primary difference in the proteolysis pattern in the presence of AdoMet is lower stability of the 42 kDa band (Fig. 2*D*). In the absence of AdoMet, the limited proteolytic signature of the M391I variant resembles that of wild-type CBS. The ∼58 kDa bands are resistant to cleavage in the K384E variant but are more susceptible in the K384N variant, forming the ∼42 kDa and additional lower MW bands (∼24 and19 kDa) (Fig. 2*C*, yellow arrowheads). AdoMet elicits only subtle changes in the variants except for stabilization of the 15 kDa band in the K384E/N and M391I variants (Fig. 2*D*, blue arrowheads). Thus, limited proteolysis analyses hint at differences in accessibility to hypersensitive trypsin cleavage sites, particularly between the K384E and K384N variants in the absence of AdoMet, as well as between them and wild-type CBS.

### The linker mutations impact CBS activity and allosteric regulation

The specific activities of the variants was assessed in the canonical serine-dependent cystathionine synthesis and the non-canonical cysteine-dependent cystathionine and H_2_S synthesis assays (Table 1). In the canonical assay, the activities of the variants were 8- (K384E/N) and 1.4-fold (M391I) lower than wild-type CBS. In the H_2_S synthesis assay, the activities of the variants in the absence of AdoMet, were 10- (K384E) or ∼3-fold (K384N, M391I) lower than wild-type CBS. The *K*_M_ values for cysteine (*K*_M1_ = 7 ± 1 mM, and *K*_M2_ = 25 ± 4 mM) for the three variants were identical within experimental uncertainty, to that of wild-type CBS (*K*_M1_ = 6.8 ± 1.7 mM, and *K*_M2_ = 27 ± 4 mM) (30). The *K*_M_ values for homocysteine were 6.7 ± 2.6 mM (K384E), 3.3 ± 0.3 mM (K384N), and 3.8 ± 0.7 mM (for M391I) CBS, similar to the value for the wild-type enzyme (3.2 ± 1.3 mM) (30).

**Table 1:** Comparison of L-serine-dependent cystathionine and cysteine-dependent H_2_S synthesis specific activities by wild-type and linker variants of human CBS^a^

CBS	Cyst^b^ synthesis (μM mg^−1^h^−1^)		H_2_S synthesis (μM mg^−1^h^−1^)	
	AdoMet: −	+	AdoMet: −	+
Wild-type	127 ± 22	313 ± 44	726 ± 14	1512 ± 12
K384E	16 ± 3	16 ± 3	72 ± 9	45 ± 3
K384N	17 ± 2	7 ± 2	240 ± 33	156 ± 20
M391I	89 ± 7	80 ± 13	277 ± 56	337 ± 40

Serine + Homocysteine → Cystathionine + H_2_O [1].

Cysteine + Homocysteine → Cystathionine + H_2_S [2].

a Both assays lead to cystathionine synthesis as shown in Equations 1 and 2. In the canonical serine-dependent assay, cystathionine synthesis is monitored while in the cysteine-dependent assay, H_2_S synthesis is monitored. Data in the table are the mean ± SD of 3 to 7 independent repeats.

b Cyst denotes cystathionine.

In the presence of AdoMet, wild-type CBS showed a 2.5-fold increase in activity in the canonical assay. In contrast, the K384N variant exhibited a 2.5-fold decrease while the K384E variant was unresponsive to AdoMet. In the H_2_S assay, AdoMet elicited a 2-fold increase in wild-type CBS activity, but an ∼1.5-fold decrease with the K384E/N variants (Table 1). The activity of the M391I variant was unresponsive within experimental uncertainty to AdoMet, in both assays. Due to the limited availability of M391I CBS and its tendency to precipitate in the presence of high serine concentrations, further characterization was focused on K384E/N CBS.

### K_Dapp_ for serine is sensitive to CBS concentration

**Figure 3:**
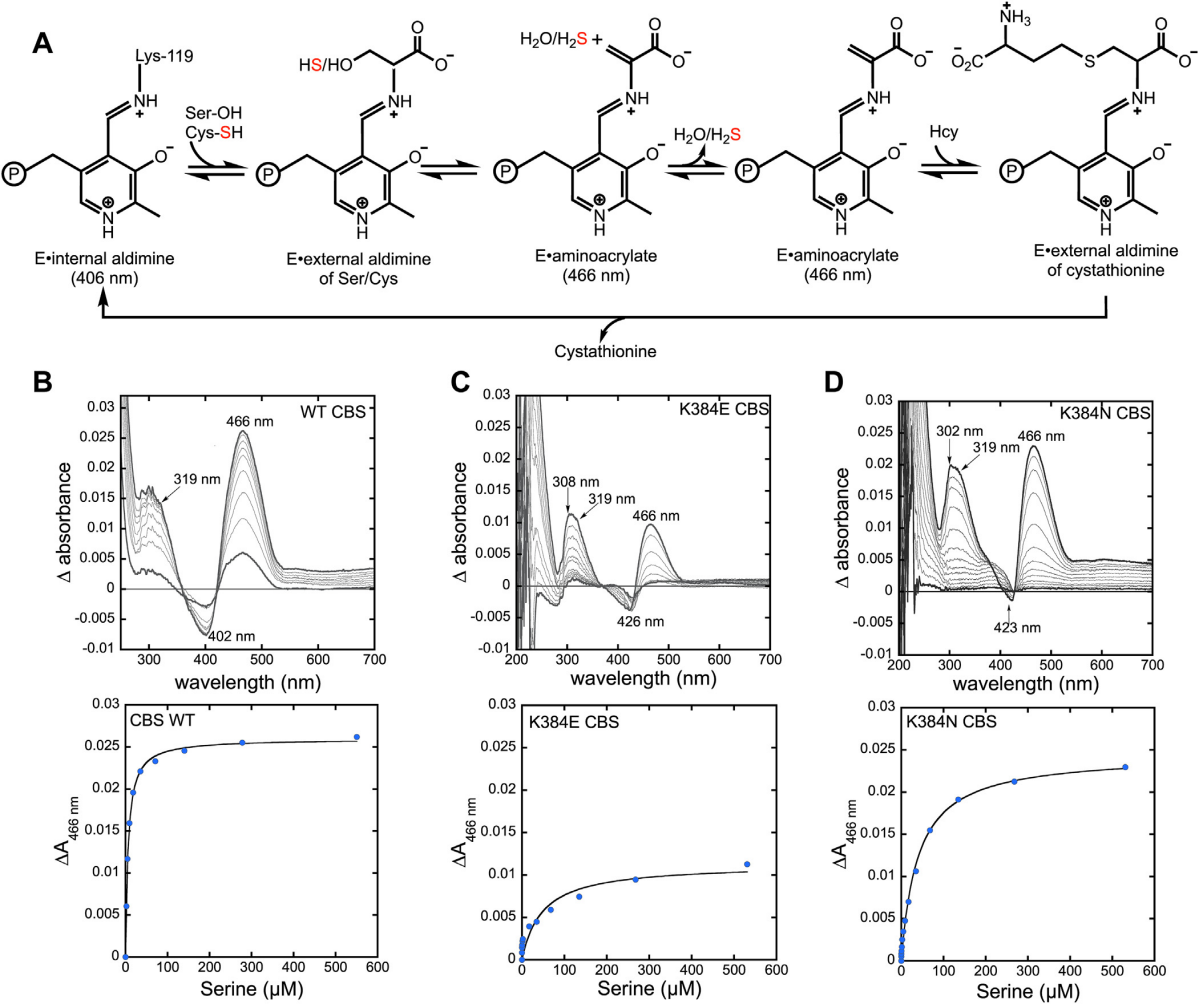
**CBS reaction mechanism and serine binding.***A*, scheme showing key PLP-based intermediates formed during CBS-catalyzed cystathionine formation from cysteine or serine, which leads to the elimination of sulfide or water, respectively. *B*–*D*, difference absorption spectra were obtained upon adding increasing concentrations of serine (2, 5, 9, 18, 36, 71, 140, 278, and 550 μM) to 4 μM wild-type (*B*), K394E (*C*) or K384N (*D*) CBS in 20 mM HEPES pH 7.4 at 25 °C. The *lower panels* in *B*–*D* show the dependence of Δ466 nm on serine concentration.

Detection of PLP-bound intermediates in the CBS-catalyzed reaction (Fig. 3*A*) is complicated by the intense and overlapping Soret band (at 428 nm) of the heme cofactor. To circumvent this limitation, reaction intermediates have been previously characterized with a variant lacking the N-terminal heme-binding site, which retains only 40% of wild-type activity (31), or by difference stopped-flow spectroscopy, using full-length CBS (32). We used difference absorption spectroscopy to monitor the reaction of serine with the K384E/N variants at two concentrations of CBS (Table 2). With wild-type CBS, a signature increase in absorption at 466 nm and decrease at 402 nm was observed (Fig. 3*B*), which were previously assigned to the aminoacrylate and internal aldimine, respectively (25, 32). The increase in absorption at ∼319 nm was previously assigned to the enolimine tautomer of the aminoacrylate intermediate (32, 33). While an increase at 466 nm is also observed with the K384E/N CBS, unlike the wild-type enzyme, the variants show a decrease in absorbance at ∼425 nm rather than at 406 nm, suggesting differences in the PLP electronic environment between the resting enzymes (Fig. 3, *C* and *D*). In addition, the ratio of the 319:466 nm bands is higher in the K384E/N variants, implying a higher concentration of the inactive enolimine tautomer of PLP. The 466 nm aminoacrylate intermediate is 1.4-fold lower in intensity in the K384E variant compared to wild-type CBS at the same concentration of serine. From the dependence of ΔA_466nm_ on serine concentration, *K*_Dapp_ values of 7 ± 1 μM (wild-type), 48 ± 19 (K384E) and 40 ± 4 μM (K384N) were obtained at the lower (4 μM) protein concentration (Fig. 3, *B*–*D*, lower panels). We next assessed whether the serine *K*_Dapp_ is affected by the concentration-dependent propensity of full-length CBS to shift to higher-order oligomers (Fig. 2*B*). While wild-type and K384N CBS exhibited 6- and 3-fold increases at the higher protein concentration (15 μM), the *K*_Dapp_ for serine decreased 2-fold for the K384E variant (Table 2). The reason for the differential effects of protein concentration on *K*_Dapp(Ser)_ is currently not understood.

**Table 2:** Comparison of apparent *K*_*D*_ for L-serine binding at two concentrations of CBS

CBS	*K*_Dapp_, μM	*K*_Dapp_, μM
	4 μM CBS	15 μM CBS
Wild-type	7 ± 1	44 ± 9
K384E	48 ± 19	24 ± 2
K384N	40 ± 4	107 ± 13

### AdoMet enhances the rate of serine binding and aminoacrylate formation

**Figure 4:**
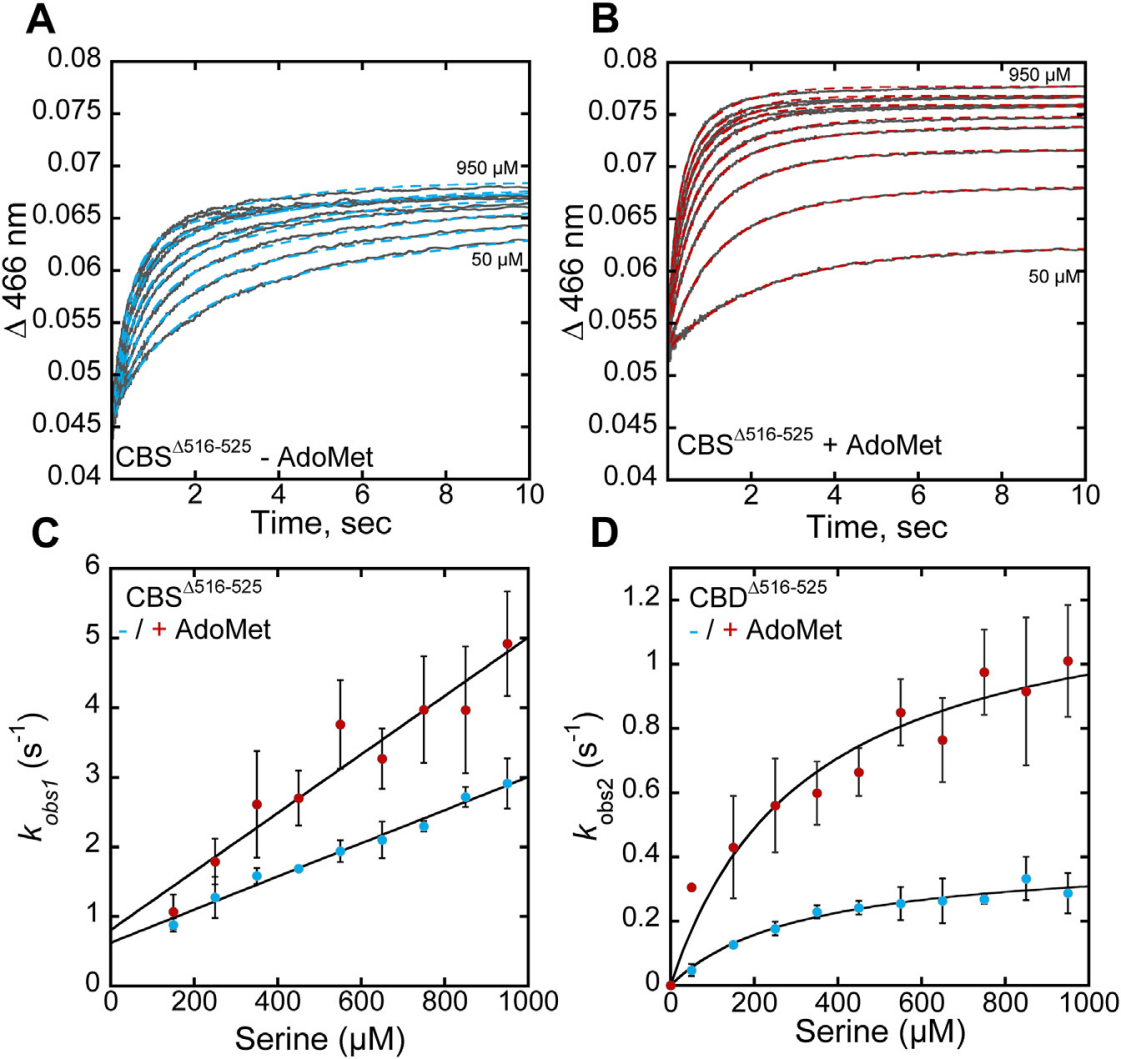
**Reaction of serine with CBS**^**Δ516–525**^**variants.***A* and *B*, representative traces for serine binding to CBS^Δ516–525^ in 20 mM HEPES pH 7.4, containing 0.5 mM TCEP, without (*A*) and with 380 μM AdoMet (*B*). Serine concentrations were varied from 50 to 950 μM and the traces were monitored at 466 nm, which reports on aminoacrylate formation. *C* and *D*, the dependence of the first (*k*_obs1_) (*C*) and second (*k*_obs2_) (*D*) phases on serine concentration in the absence (*blue*) and presence (*red*) of 380 μM AdoMet. The *K*_Dapp_ values for serine estimated from the data in *panel D* are: 316 ± 97 μM (−AdoMet) and 324 ± 67 μM (+AdoMet). Each data point represents the mean ± SD of at least two independent experiments with at least two technical replicates.

The quaternary structure of recombinant CBS as isolated comprises a mixture of 2- and 4-mers as well as oligomers organized as filaments of variable length (27). To circumvent the potentially confounding effects of mixed oligomeric states on kinetic analyses, we examined the kinetics of aminoacrylate formation with the K384E/N variants in the CBS^Δ516–525^ background, which stabilizes the dimer (14, 24). The reaction of serine with CBS^Δ516–525^ ± AdoMet displays biphasic kinetics (Fig. 4, *A* and *B*). From the linear dependence of the first phase (*k*_*obs1*_) on serine concentration, values for *k*_*on*_ (2380 ± 100 M^−1^ s^−1^) and *k*_off_ (0.6 ± 0.07 s^−1^) at 20 °C were estimated in the absence of AdoMet (Fig. 4*C*, Table 3). The hyperbolic dependence of the second phase on serine yielded a maximal rate (*k*_*obs2*_) of 0.4 ± 0.03 s^−1^ (Fig. 4*D*). In the absence of AdoMet, the amplitude changes associated with *k*_obs1_ and *k*_obs2_ increased and decreased, respectively as serine concentration was increased (Fig. S1*A*).

**Table 3:** Comparison of rate constants for L-serine binding and aminoacrylate intermediate formation for the K384E/N variants in the CBS^Δ516–525^ background^a^

Enzyme	*k*_on_, M^−1^s^−1^		*k*_off_, s^−1^		b*k*_*obs2*_, s^−1^	
AdoMet	−	+	−	+	−	+
CBS (full-length)	2245 ± 153	4776 ± 278	0.1 ± 0.1	0.5 ± 0.2	0.6 ± 0.2	1.7 ± 0.3
CBS^Δ516–525^	2380 ± 110	4150 ± 450	0.6 ± 0.07	0.9 ± 0.3	0.4 ± 0.03	1.4 ± 0.1
K384N-CBS^Δ516–525^	30 ± 4	31 ± 2	0.09 ± 0.04	0.06 ± 0.02	0.07 ± 0.01	0.15 ± 0.02
K384E-CBS^Δ516–525^	26 ± 1	25 ± 1	0.005 ± 0.0005	0.014 ± 0.0006	cNA	NA

a The data represent the mean ± SD from at least two independent experiments each with at least two technical replicates.

b *k*_obs2_ refers to the rate constant assigned to the dissociation of water from the E•aminoacrylate•H_2_O intermediate in the active site.

c NA refers to not available since the second phase was not observed with this variant.

We tentatively assign *k*_obs1_ to formation of the ternary E•aminoacrylate•H_2_O product complex. The linear dependence of *k*_obs1_ on serine indicates that serine binding is slow relative to the subsequent steps leading to aminoacrylate intermediate formation, which occurs more rapidly. We tentatively assign *k*_obs2_ to the formation of the binary E•aminoacrylate complex, formed upon loss of H_2_O from the active site, as a prelude to homocysteine binding (Fig. 3*A*). The residual amplitude of the second phase indicates that formation of the ternary E•aminoacrylate•H_2_O complex does not go to completion even at the highest concentration of serine used in this study. The equilibrium between the internal aldimine and the ternary complex also includes the external aldimine intermediate (see Fig. 3*A*), which does not absorb at 466 nm. As serine concentrations increase, the ternary complex is pulled in the forward direction to the spectrally indistinguishable (at 466 nm) binary E•aminoacrylate intermediate, accounting for the two phases. The hyperbolic dependence of *k*_obs2_ on serine concentration indicates that the loss of H_2_O from the active site is likely accompanied by a protein conformational change, which is rate limiting (*k*_max_ = 0.4 ± 0.03 s^−1^). PLP enzymes are known to undergo conformational changes that are sensitive to substrate binding/product release. A substrate-triggered open to closed conformational change has been seen in the crystal structures of *Drosophila* CBS (25).

AdoMet increased *k*_*on*_ 1.7-fold (4150 ± 450 M^−1^ s^−1^) and *k*_obs2_ 3.5-fold (to 1.4 ± 0.1 s^−1^) while *k*_off_ (0.9 ± 0.3 s^−1^) was largely unaffected within experimental uncertainty (Fig. 4, *C* and *D*). The amplitude changes associated with *k*_obs1_ and *k*_obs2_ was largely independent of serine concentration in the presence of AdoMet (Fig. S1*B*). Full-length CBS showed comparable modulation of these kinetic parameters by AdoMet (Table 3), revealing that the oligomeric state does not influence the reaction at least up to the E•aminoacrylate intermediate, and, that AdoMet enhances *k*_on(Ser)_ and *k*_*obs2*_ on the order of 2 to 3.5 fold.

**Figure 5:**
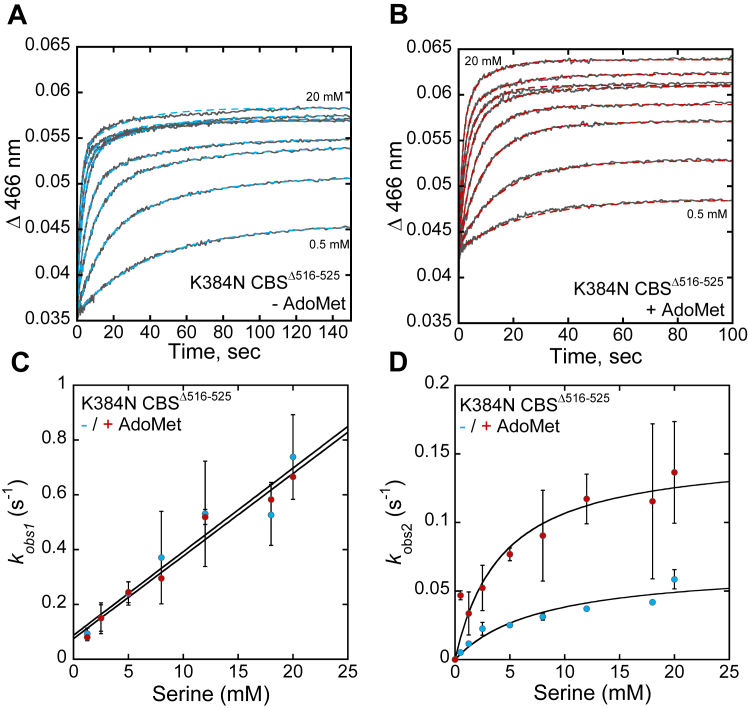
**Reaction of L-serine with K384N CBS**^**Δ516–525**^**.***A* and *B*, representative traces for serine binding to K384N CBS^Δ516–525^ in 20 mM HEPES pH 7.4, 0.5 mM TCEP, without (*A*) and with 380 μM AdoMet (*B*). The L-serine concentration was varied from 0.5 to 20 mM. The traces were monitored at 466 nm, which corresponds to aminoacrylate formation. *C* and *D*, dependence of *k*_*obs1*_ (*C*) and *k*_*obs2*_ (*D*) on serine concentration for K384N CBSΔ^516–525^ in the absence (*blue*) and presence (*red*) of 380 μM AdoMet. The *K*_Dapp_ values for serine estimated from the data in *panel D* are: 8 ± 3 mM (-AdoMet) and 4 ± 1 mM (+AdoMet). Each data point represents the mean ± SD of at least two independent experiments with at least two technical replicates.

K384N CBS^Δ516–525^ also displayed biphasic kinetics in the presence or absence of AdoMet (Fig. 5, *A* and *B*). The amplitude changes associated with each phase showed a similar sensitivity to serine concentration as seen with CBS^Δ516–525^ (Fig. S1, *C* and *D*). The *k*_on_ (30 ± 4 M^−1^ s^−1^) and *k*_off_ (0.09 ± 0.04 s^−1^) values derived from the first phase were 80- and 7-fold smaller than for CBS^Δ516–525^, and AdoMet had no effect on either parameter (Table 3, Fig. 5*C*). The rate constant for the second phase *k*_obs2_ (0.07 ± 0.01 s^−1^), was 6-fold smaller than for CBS^Δ516–525^, and increased ∼2-fold (0.15 ± 0.02 s^−1^) in the presence of AdoMet (Fig. 5*D*). Hence, while the K384N mutation renders *k*_on(Ser)_ insensitive to AdoMet, the subsequent step (*k*_obs2_) retains sensitivity.

**Figure 6:**
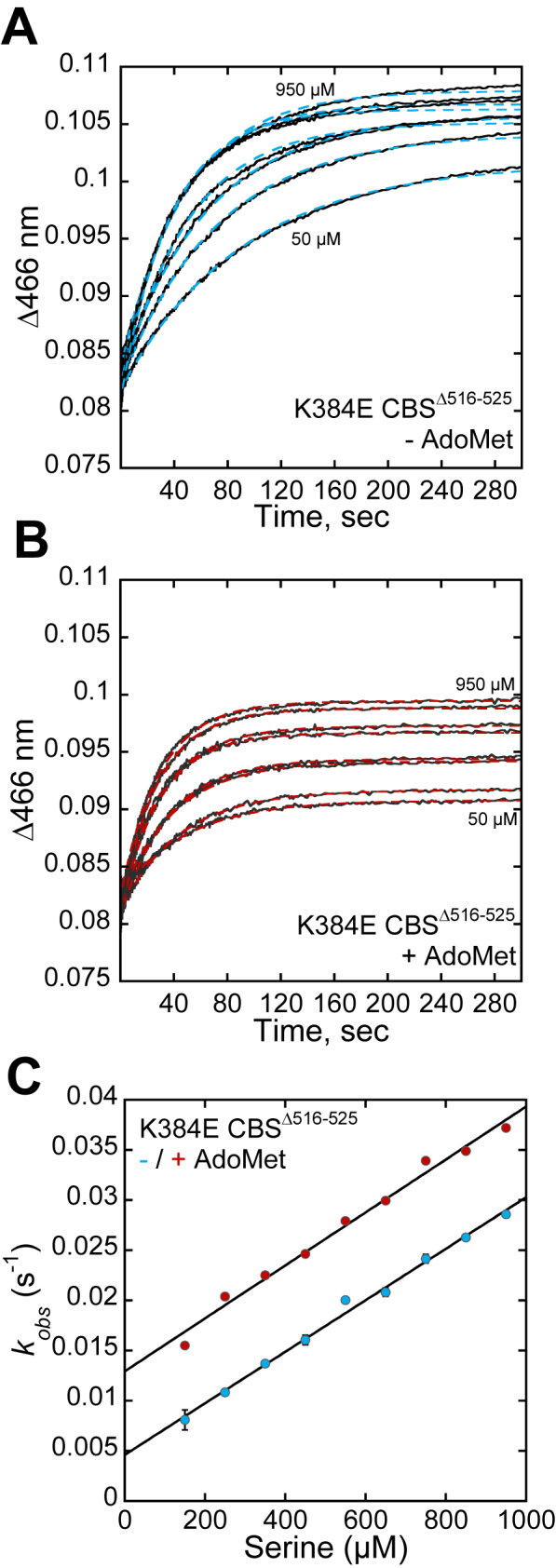
**Reaction of L-serine with K384E CBS**^**Δ516–525**^**.***A* and *B*, representative traces for serine binding to K384E CBS^Δ516–525^ in 20 mM HEPES pH 7.4 containing 0.5 mM TCEP, without (*A*) and with 380 μM AdoMet (*B*). The L-serine concentration was varied from 50 to 950 μM. The traces were monitored at 466 nm, which corresponds to aminoacrylate formation. *C*, dependence of *k*_obs_ on serine concentration in the absence and presence of AdoMet. Each data point represents the mean ± SD of at least two independent experiments with at least two technical replicates.

The K384E variant exhibited monophasic kinetics, suggesting a change in the component rate constants (Fig. 6*A*). From the linear dependence of *k*_obs_ (at 466 nm) on serine concentration (Fig. 6*B*), the *k*_on_ was estimated to be 26 ± 1 M^−1^ s^−1^, which was unresponsive to AdoMet. The *k*_off_ for serine (0.005 ± 0.0005 s^−1^) increased ∼3-fold to 0.014 s^−1^ in the presence of AdoMet; however, both values were too low to be reliably determined. The change in amplitude increased with increasing concentration of serine (Fig. S2, *A* and *B*).

### Kinetics of homocysteine-dependent aminoacrylate intermediate disappearance

**Figure 7:**
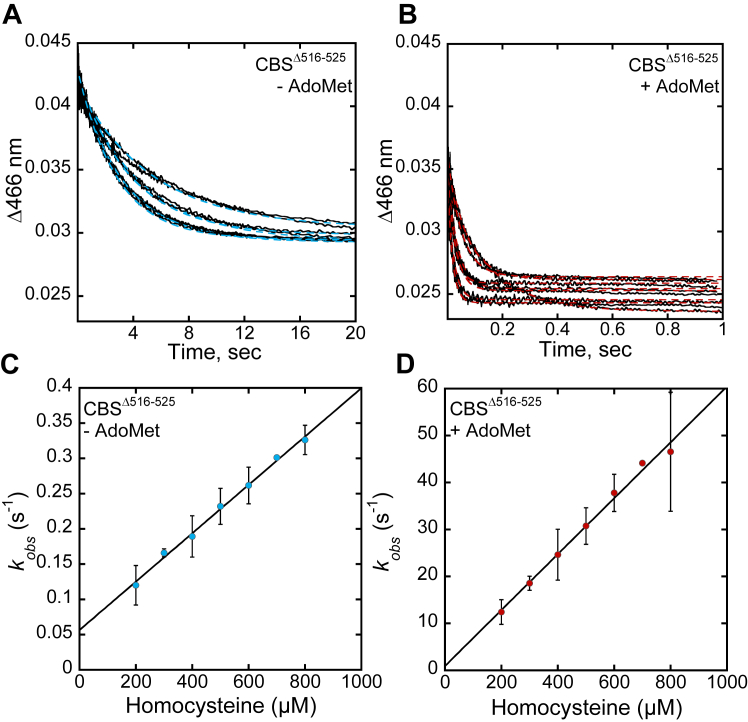
**Reaction of homocysteine with the aminoacrylate intermediate on CBS**^**Δ516–525**^**.***A* and *B*, representative traces for homocysteine-dependent loss of the aminoacrylate intermediate upon mixing varying concentrations of homocysteine (200–800 μM) with 3 μM CBS^(Δ516–525)^ in 20 mM HEPES pH 7.4, containing 0.5 mM TCEP, pretreated with 80 μM serine, without (*A*) and with 380 μM AdoMet (*B*). *C* and *D*, the dependence of *k*_*obs*_ on homocysteine concentration in the absence (*C*) and presence (*D*) of AdoMet. Each data point represents the mean ± SD of at least two independent experiments with at least two technical replicates.

The influence of AdoMet on the second half reaction, that is, addition of homocysteine to the aminoacrylate intermediate, forming cystathionine (Fig. 3*A*), was examined next. For this, varying concentrations of homocysteine were rapidly mixed with CBS^Δ516–525^, which had been pre-incubated with 80 μM serine ± 380 μM AdoMet (Fig. 7, *A* and *B*). In the presence of AdoMet, the change in amplitude at 466 nm was approximately two-thirds of that observed in its absence, suggesting a change in the relative proportion of the aminoacrylate and downstream species (*e.g.* external or internal aldimine). CBS^Δ516–525^ exhibited monophasic kinetics with a *k*_*obs*_ of 340 ± 10 M^−1^ s^−1^, which increased to 59,500 ± 2300 M^−1^ s^−1^ in the presence of AdoMet (Table 4, Fig. 7, *C* and *D*). These values were comparable to those for full-length CBS (Table 4). We ascribe this complex rate constant to the steps leading from homocysteine binding through its reaction with the aminoacrylate species.

**Table 4:** Rate constants for the reaction of homocysteine with the aminoacrylate intermediate for linker variants in the CBS^Δ516–525^ background^a^

Enzyme	b*k*_*(aa→Cyst)*_, M^−1^ s^−1^	
	−AdoMet	+ AdoMet
CBS (full-length)	250 ± 4	52,600 ± 2100
CBS^Δ516–525^	340 ± 10	59,500 ± 2300
K384N-CBS^Δ516–525^		
major	320 ± 20	cNA
minor	9900 ± 1100	2350 ± 400
K384E-CBS^Δ516–525^		
major	70 ± 1	NA
minor	1600 ± 200	1700 ± 100

a The data represent the mean ± SD from at least two independent experiments each with at least two technical replicates. The *k*_off_ values were too low to be reliably estimated.

b *k*_*(aa→Cyst)*_ for denotes the lumped rate constant for steps including homocysteine binding and reaction with the aminoacrylate intermediate. The rate constants for the major and minor species of the K384E/N variants correspond to the amplitude changes associated with each phase.

c NA denotes not applicable.

**Figure 8:**
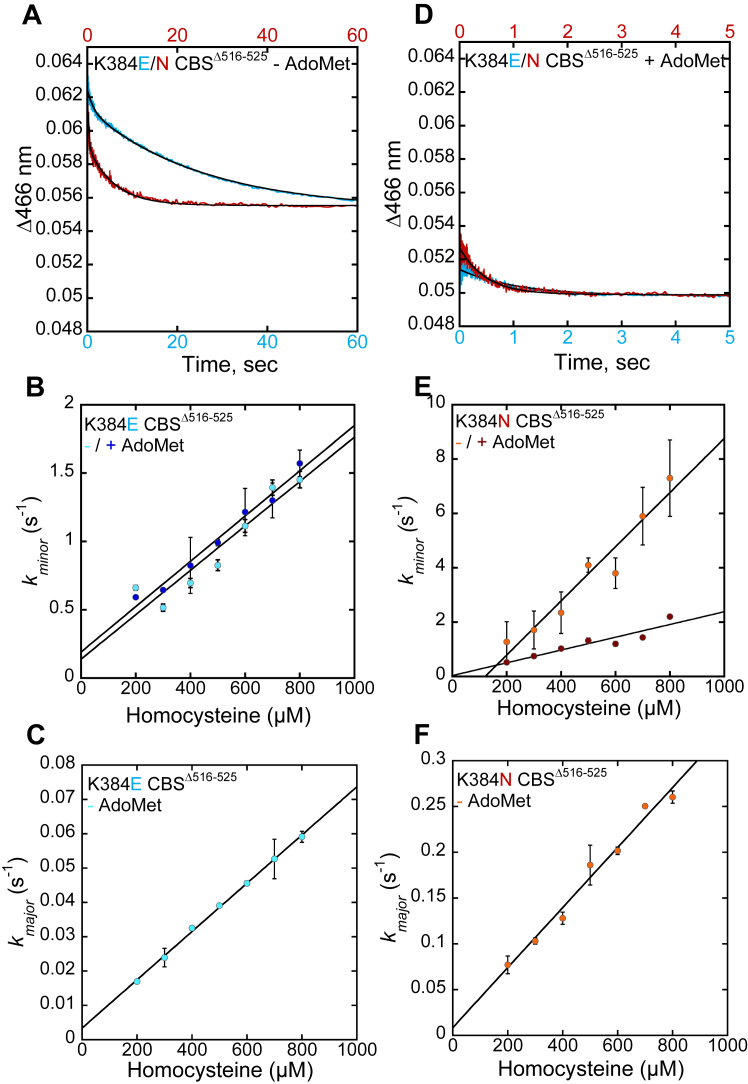
**Homocysteine binding to K384E/N CBS**^**D516–525**^**variants.***A* and *D*, representative stopped-flow trace for 500 μM homocysteine reacting with the aminoacrylate intermediate on 4 μM K384E (blue trace) CBS^Δ516–525^ or 5 μM K384N (red trace) CBS^Δ516–525^ premixed with 80 μM L-serine in 20 mM HEPES, 0.5 mM TCEP pH 7.4 in the absence (*A*) and presence (*D*) of 380 μM AdoMet. *B*, the dependence of *k*_*minor*_ for the K384E variant on homocysteine concentration in the absence and presence of AdoMet. *C*, the dependance of *k*_*major*_ for the K384E variant on homocysteine concentration in the absence of AdoMet. *E*, the dependence of *k*_*minor*_ for the K384N variant on homocysteine concentration in the absence and presence of AdoMet. *F*, the dependance of *k*_*major*_ for the K384N variant on homocysteine concentration in the absence of AdoMet Each data point represents the mean ± SD of at least two independent experiments and with at least two technical replicates.

The K384E/N CBS^Δ516–525^ variants exhibit biphasic kinetics in the absence of AdoMet with both phases showing a linear dependence on homocysteine concentration (Fig. 8*A*). We interpret the two phases as evidence for a minor and a major population (∼1:1.5 ratio), based on the amplitude changes in the first and second phases, respectively. The rate constants for the minor populations were 9900 ± 1100 M^−1^ s^−1^ (K384N) and 1600 ± 200 M^−1^ s^−1^ (K384E) and for the major population were 320 ± 20 M^−1^ s^−1^ (K384N) and 70 ± 1 M^−1^ s^−1^ (K384E) (Fig. 8, *B*, *C*, *E*, and *F*, Table 4). The total amplitude change observed with the K384E/N CBS^Δ516–525^ variants was ∼50% of that seen with CBS^Δ516–525^.

In the presence of AdoMet, monophasic kinetics were observed, albeit of very low amplitude (Fig. 8*D*). Replots of the dependence of *k*_obs_ on homocysteine concentration yielded values of 1700 ± 100 M^−1^ s^−1^ (K384E) and 2350 ± 400 M^−1^ s^−1^ (K384N) CBS^Δ516–525^ (Fig. 8, *B* and *E*). Thus, AdoMet elicited a 4-fold decrease with K384N CBS^Δ516–525^, but had no effect on the K384E variant. Importantly, under these conditions, only a third of the total population of either variant responded to homocysteine at rates that were 25- (K384N) and 35-fold (K384E) lower than CBS^Δ516–525^. Only the minor population of the K384E/N variants appeared to be responsive to AdoMet.

### Crystal structure of truncated K384N CBS

**Figure 9:**
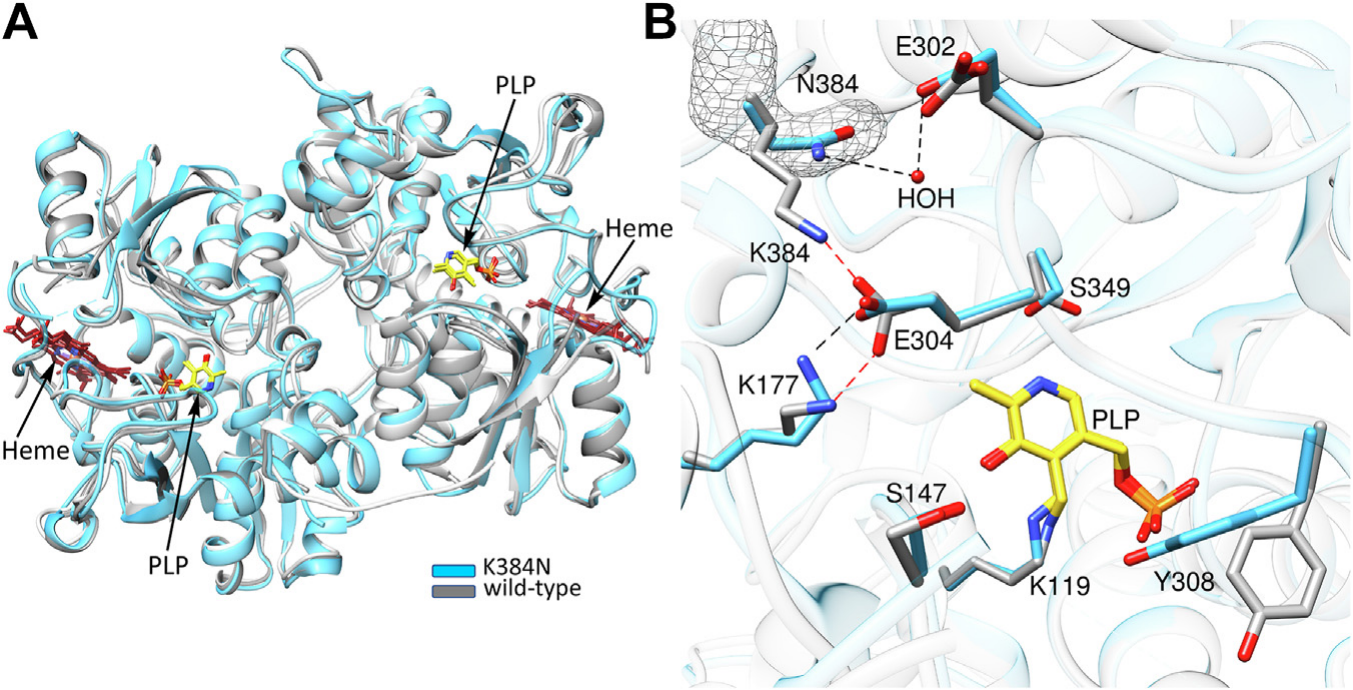
**Crystal structure of K384N**^**Δ411–551**^**CBS.***A*, an overlay of CBS^Δ411–551^ (*gray*) (PDB code: 1JBQ) on K384N CBS^Δ411–551^ (*blue*) reveals no major changes in the overall structures. The PLP and heme cofactors are shown in *yellow* and *red* stick displays, respectively. *B*, close-up of the active site shows that the PLP (*yellow sticks*) binding pocket is unperturbed by the K384N mutation. Salt bridges between Glu-304 and Lys-384 or Lys-177 in CBS^Δ411–551^ (*gray sticks*) are shown as *red dashed lines*. Hydrogen bonds between Asn-384, bridging water, and Glu-302 (*blue sticks*) are shown as *black dashed lines*. mFo-DFc omit map of Asn-384 at 3σ is shown as a *grey mesh*.

The shift in the equilibrium of the K384E/N variants to lower-order oligomers (Fig. 2, *A* and *B*) motivated crystallization attempts in the full-length and the CBS^Δ516–525^ backgrounds, which were however, unsuccessful. Deletion of the C-terminal regulatory domain yields a stable and active form of CBS, which crystallizes readily (34, 35). We therefore utilized this truncated CBS variant to evaluate whether the K384N mutation affects the active site architecture. The crystal structure of K384N^Δ411–551^ CBS was solved by molecular replacement at 2.4 Å resolution (Table 5), using a previously solved structure of CBS^Δ411–551^ (PDB code: 1JBQ). An alignment of the K384N^Δ411–551^ structure with two CBS^Δ411–551^ structures, showed that the K384N mutation did not elicit major changes overall (Cα RMSD of 0.417 Å *versus* PDB code: 1JBQ and 0.570 Å *versus* PDB code: 1M54) (Fig. 9*A*). A close-up of the active site showed that the most notable change was the expected loss of a salt bridge between Lys-384 and Glu-304 (Fig. 9*B*). In the structure of wild-type CBS^Δ411–551^, Glu-304 forms a salt bridge with Lys-177, which is retained in the K384N variant. Asn-384 forms hydrogen bonds with Glu-302 *via* a bridging water, while the position of Glu-302 is the same in the two structures. The PLP binding pocket appears to be unperturbed by the K384N mutation, with the exception of the Tyr-308 side chain, which is rotated towards PLP. Tyr-308 is analogous to Tyr-277 in *Drosophila* CBS, and was proposed to stabilize the carbanion intermediate as well as to activate the hydroxyl leaving group on serine (25). While it is possible that the K384N mutation impacts protein conformational dynamics between the basal and activated states (Fig. 1, *C* and *D*), substantial differences were not observed in the truncated CBS ^Δ411–551^ protein form.

**Table 5:** Data collection and refinement statistics

K384N CBS^Δ411–551^	
Data Collection	
Beamline	APS, LS-CAT-F
Wavelength (Å)	0.976
Temperature (K)	100
Space group	P 1
Cell dimension	
α, β, γ (º)	89.46, 90.19, 104.22
a, b, c (Å)	106.15, 105.49, 107.47
Resolution (Å)	43.07 (2.4)^a^
*R*_merge_(%)	7.8 (63)
*R*_meas_(%)	9.0 (73)
*R*_pim_(%)	4.6 (40)
<I/σ>	8.1 (1.6)
CC (½)	0.997 (0.993)
Completeness (%)	93.3 (95.3)
Multiplicity	3.8 (3.9)
No. Reflections	387,521 (19,984)
No. Unique Reflections	100,667 (5138)
Refinement	
Resolution Range	30.75 (2.4)
Number of reflections (work/test)	99,538/4866
*R*_work_ /*R*_free_ (%)	21.04/24.3
No. of atoms	
protein	2783
water	32
Ligands: PLP	90
Heme	252
B-factors(Å^2^)	
Protein	68.7
Ligands	53.9
Water	54.9
Rmsd deviations	
Bond lengths (Å)	0.009
Bond angles (º)	1.664
Ramachandran plot (%)	
Favored, allowed, outliers	95.42, 4.06, 0.52
MolProbity score	1.74
PDB code	8STW

a Values in parentheses are for highest-resolution shell.

## Discussion

New structures of the native fibrillar forms of CBS have provided insights into a potential mechanism for allosteric regulation by AdoMet (27). In the basal state, the interfaces between the adjacent units are built by the crossed-over regulatory domains of the CBS dimers, and the lower activity of the basal state is attributed to impeded active site access (Fig. 1*E*). AdoMet triggers and/or stabilizes a dramatic reorganization. While the regulatory domains from adjacent dimers continue to build interfaces, the catalytic cores protrude from the central fiber, enhancing access to substrates. Patient mutations in the linker region of CBS, which had not been biochemically characterized previously, are predicted to affect allosteric regulation by AdoMet. Despite the >50 Å distance between the heme- and C-terminal domains in the activated conformation, AdoMet increases the affinity of the CBS ferrous heme for NO• (2-fold) and CO (5-fold) (36). The important role of the regulatory domain in fibril organization thus provides a structural framework for understanding long-range communication with the heme cofactor, which could be transmitted across dimer interfaces rather than within a single dimeric unit.

All three linker variants characterized in this study have lower activity than wild-type CBS (Table 1). Since the specific activity of CBS is ∼6-fold higher for H_2_S synthesis *versus* the canonical serine consumption reaction, the low activity variants are more reliably assessed in the H_2_S synthesis assay. There are differences in the magnitude by which two of the linker variants (K384N and M391I) impact the canonical *versus* H_2_S synthesis reactions, a phenomenon that has been seen previously with mutations in the catalytic core (21). In the absence of AdoMet, the K384N mutation decreases the canonical and H_2_S synthesis activities by 90% and 70%, while the M391I mutation decreases the same activities by 30% and 62%, respectively (Table 1). On the other hand, the K384E mutation decreases both activities by ∼90%. The differential effects of the K394N and M391I mutations on two similar β-elimination reactions might reflect how each residue impacts the leaving group potential of H_2_S *versus* H_2_O, respectively (Fig. 3*A*). In combination with lower protein stability, it is possible that the significantly lower clearance of homocysteine *via* the H_2_S synthesis rather than the canonical reaction, contributes to homocysteine accumulation in patients harboring the M391I mutation.

Differences in activity between wild-type CBS and the variants are exacerbated by AdoMet, which either fails to elicit an effect (M391I) or inhibits activity (K384N). Curiously, AdoMet inhibits H_2_S synthesis but has no effect on the canonical activity of K384E CBS (Table 1). NO and CO are other known inhibitors of CBS that interact with the heme cofactor (9, 11), and their potency increases in the presence of AdoMet, while nitrite leads to an inhibitory ferrous-nitrosyl species (12). It is curious that the K384E/N linker mutations flip the AdoMet effect from activation to inhibition, a phenomenon that has also been seen with the T257I/M mutations located in the catalytic core (21). While the K384E/N variants show a decreased propensity for forming higher order oligomers, it is unclear whether they also impair the conformational shift from the basal to the activated state. Loss of AdoMet regulation in M391I CBS is not due to its inability to bind the ligand since AdoMet clearly influences the limited proteolysis pattern (Fig. 2, *C* and *D*).

**Figure 10:**
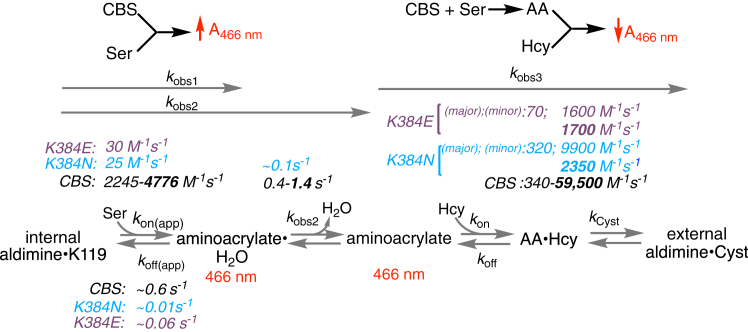
**Summary of the pre-steady state kinetic analysis of CBS linker mutants.** Comparison of the pre-steady kinetic behavior of variants in the CBS^Δ516–525^ background. The values depicted in bold lettering were obtained in the presence of AdoMet. AA denotes the aminoacrylate intermediate.

The presence of 2- and 4-mers mixed with native fibers of varying lengths complicates kinetic and thermodynamic analyses of CBS. Therefore, the K384E/N mutations were introduced in the CBS^Δ516–525^ background to stabilize the dimer (14). Kinetic analyses provided insights into the penalties associated with the K384E/N linker variants in the absence and presence of AdoMet (Fig. 10). It is important to note that the rate constants for the formation and disappearance of the aminoacrylate intermediate are complex and each includes multiple steps. For example, *k*_obs1_ for aminoacrylate formation includes steps describing the serine on and off rates in addition to the chemical steps through the *gem*-diamine, external aldimine, and carbanion intermediates, which are unresolved spectrally, and therefore, kinetically. With CBS^Δ516–525^, AdoMet enhances *k*_on_ for serine 2-fold, while it enhances the lumped rate constant for homocysteine-dependent aminoacrylate disappearance 175-fold (*i.e. k*_Cyst_) (Fig. 10). Hepatic serine concentration is estimated to vary from ∼0.4 to 0.8 mM (37, 38, 39). Based on a *K*_Dapp_ for serine of ∼7 μM, CBS is predicted to exist predominantly in the aminoacrylate intermediate state. Our kinetic analysis predicts that when the cell is replete with methionine, AdoMet accelerates homocysteine binding and consumption 175-fold, committing sulfur to the transsulfuration pathway.

The K384E/N mutations significantly impact *k*_on(app)_ for serine, which is unresponsive to AdoMet in contrast to CBS^Δ516–525^. In the absence of AdoMet, the kinetic behavior of the aminoacrylate intermediate on K384E/N CBS^Δ516–525^ is interpreted as reporting on a major and a minor population. While the major population displays modest (K384E) or no (K384N) impact on *k*_obs3_, the minor population shows a 5- (K384E) or 7-fold (K384N) faster rate of aminoacrylate disappearance than CBS^Δ516–525^ (Fig. 10). AdoMet only affects the minor population of the K384N variant but has no effect on K384E CBS.

Our pre-steady state kinetic analysis reveals that the K384E/N substitutions equally impact the steps leading up to aminoacrylate intermediate formation, while the differences between them are expressed in *k*_*obs3*_, *i.e.* resolution of the intermediate (Fig. 10). Both substitutions shift the equilibrium away from the higher order oligomers toward the 2- to 4-mers (Fig. 2*A*), supporting the functional importance of the fibers, particularly for AdoMet-dependent regulation. On the other hand, since AdoMet activation of the dimeric CBS^Δ516–525^ is comparable to that of wild-type enzyme, it suggests that the linker mutations exert additional effects on conformational toggling between the basal and activated states, which is consistent with altered access to hypersensitive cleavage sites revealed by limited proteolysis (Fig. 2, *C* and *D*). In summary, the study reveals that clinical variants in the linker region impact CBS oligomerization, which is correlated with changes in activity as well as AdoMet-dependent regulation.

## Experimental procedures

### Materials

Amicon Ultra 15 centrifugal filters, D,L-homocysteine, L-serine, sodium phosphate, trifluoroacetic acid (TFA), tris(hydroxymethyl)aminomethane (Tris), and 2-[4-(2-hydroxyethyl)piperazin-1-yl]ethanesulfonic acid (HEPES) were all purchased from Sigma. Ammonium hydroxide, hydrochloric acid, and sodium dodecyl sulfate were purchased from Fisher. Ethylenediaminetetraacetic acid (EDTA), imidazole, and lead acetate were purchased from Acros Organics. [^14^C]-serine was from PerkinElmer. Tris (2-carboxyethyl) phosphine (TCEP) was from GoldBio. S-adenosyl-L-methionine (the disulfate tosylate salt) was purchased from BioVision. Trypsin was obtained from the Hampton Research Proti-ACE kit. EcoLite scintillation cocktail was obtained from MP Biomedicals. AG-50W-X8 anion exchange packing material was obtained from Bio-Rad. The Oligos for site directed mutagenesis were purchased from IDT. Luria Bertani (LB) broth was purchased from Research Products International (RPI).

### Expression and purification of CBS^Δ516–525^

Human CBS carrying a 10 amino acid deletion (residues 516–525) in the regulatory domain was purchased from GenScript and cloned into the pET28b+ vector incorporating a cleavable C-terminal 6-His tag. Mutations in the linker region of CBS in this background were incorporated *via* site directed mutagenesis using the QuikChange kit. The forward primers for the three variants (mutated codon underlined) were as follows; the reverse primers had the complementary sequences.

K384E: 5′-CTACATGACCGAATTCCTGAGCGA-3′.

K384N: 5′-CTACATGACCAACTTCCTGAGCGAC-3′.

M391I: 5′-GCGACAGGTGGATTCTGCAG-3′.

BL-21 *E. coli* cells were transformed with each of the expression constructs and the recombinant variants were expressed and purified as described previously (40), with a few modifications. Briefly, 6 × 1 L of LB containing 25 μg/ml kanamycin was inoculated with 5 ml per liter of overnight culture in Luria Bertani (LB) medium. Cells were grown at 37 ^°^C until an OD_600_ of 0.6 was reached prior to lowering the temperature to 28 ^°^C. After cooling for ∼1.5 h, 100 μM each of pyridoxine hydrochloride, δ-aminolaevulinic acid, ferric chloride, and thymine were added and the incubation was continued for 20 h. Since isopropyl-β-D-1-thiogalactopyranoside decreased the yield of soluble protein, the induction step was omitted.

Cells were harvested by centrifugation at 4000*g* for 40 min prior to resuspending in the sonication buffer containing 50 mM sodium phosphate buffer, pH 7.4, 0.5 mM TCEP and 20 mM imidazole. The cells were sonicated using a Misonix instrument set at ∼40% power (∼30 W) for 2 × 3.5 min with a 1 min pause. The slurry was stirred continuously in a 250 ml stainless steel beaker kept in an ice-water bath to maximize the surface area for heat exchange. The cell lysate was centrifuged at 26,916*g* for 45 min, the pellet was discarded, and the clarified cell lysate was applied to a Ni-NTA column (10 ml Thermo Scientific) equilibrated with the sonication buffer. Then, the column was washed with 120 ml of the sonication buffer and CBS was eluted with the 50 mM sodium phosphate buffer, pH 7.4, 0.5 mM TCEP and 300 mM imidazole. Fractions (5 ml) with yellow/orange color were pooled and dialyzed against 2 L of 50 mM Tris pH 8.0, containing 0.5 mM TCEP for 2 h. Thereafter, the sample was transferred to fresh buffer to which two units of tobacco etch virus (TEV) protease was added to cleave the His tag. Following overnight dialysis at 4 ^°^C, CBS was concentrated from ∼50 ml to <5 ml using Amicon Ultra 15 centrifugal filters with a 30 kDa molecular weight cutoff. The concentrated protein was then passed through a second Ni-NTA column and the His-tag cleaved CBS eluting from the column was loaded onto a 120 ml S-200 gel filtration column (GE Healthcare, now Cytiva), equilibrated with 50 mM Tris, pH 8.0, containing 0.5 mM TCEP. CBS^Δ516–525^ eluted as a dimer and was pooled, concentrated and stored at −80 ^°^C.

### Limited proteolysis of CBS

Limited proteolysis of CBS was performed as described previously (29). Briefly, the reaction mixture (100 μl total volume) contained 16 μM CBS (wild-type or one of the linker variants) in 20 mM Tris, pH 7.4, containing 150 mM NaCl ± 380 μM AdoMet. Then, a 13.5 μl aliquot was removed and mixed with 1.5 μl of 10% SDS, which served as the undigested (time = 0) sample. To the remaining mixture, 10 μl of 0.1 mg/ml trypsin was added and incubated at 37 ^°^C. Aliquots (13.5 μl) were removed at 1, 2, 4, and 6 h, quenched with 1.5 μl of 10% SDS and frozen at −80 ^°^C. Samples were analyzed by separation on a 15% SDS PAGE gel.

### Steady state assays

Two methods were used to monitor CBS activity: (i) [^14^C]-serine-dependent cystathionine synthesis assay, and (ii) cysteine-dependent H_2_S synthesis assay, as described below.

#### [^14^C]-serine-dependent cystathionine synthesis assay

This assay monitors the incorporation of a radiolabel carbon derived from serine into cystathionine, as described (41). Briefly, a 200 μl reaction mixture containing wild-type or variant CBS (5 μg) in 100 mM HEPES pH 7.4, 30 mM serine (39,000 cpm μM^−1^) ± 380 μM AdoMet was incubated at 37 ^°^C for 5 min. The reaction was initiated with 40 mM D,L-homocysteine, incubated for 30 min and quenched with 200 μl of 10% trifluoroacetic acid, and then centrifuged for 5 min at 10,000*g*. The individual reaction mixtures (350 μl) were loaded onto 2 × 1.5 cm AG 50W-X8 anion exchange columns, and eluted with a 3 ml followed by 2 × 2 ml washes with 4 M ammonium hydroxide. Fractions (2 ml) containing radiolabeled cystathionine in the second and third wash were combined with 15 ml of EcoLite scintillation cocktail and counted. The protein concentration was increased as follows with the linker variants: 50 μg (K384N and M391I) and 100 μg (K384E) on account of their lower activity.

#### H_2_S synthesis assay

H_2_S formation was monitored as described previously (42). Briefly, a 1.8 ml cuvette contained 100 mM HEPES pH 7.4, 30 mM cysteine, 40 mM D,L-homocysteine, ± 380 μM AdoMet and 400 μM lead acetate in a final volume of 1 ml was incubated at 37 ^°^C for 5 min. The reaction was initiated with CBS (1 μg of wild-type and 5–10 μg of the linker variants) and monitored at 390 nm for lead sulfide formation. The concentration of sulfide produced was estimated using an extinction coefficient of 5500 M^−1^ cm^−1^.

### Determination of serine K_dapp_

The spectrum of wild-type or variant enzymes in the full-length or CBS^Δ516–525^ background (4 μM or 15 μM based on a monomer molecular mass of 63 kDa) in 1 ml of 20 mM HEPES pH 7.4, and 0.5 mM TCEP at 20 °C, was recorded between 240 and 700 nm at a scan rate of 60 nm/s and 0.5 nm intervals. Aliquots of serine (to give final concentrations of 2, 5, 9, 18, 36, 71, 140, 278, and 550 μM) were added to the reaction mixture and spectra were recorded following 3-min incubation periods to allow for equilibration. Difference spectra were processed after correcting for dilution, using the KaleidaGraph software. The change in absorbance at 465 nm was used to determine fractional saturation, which was plotted against the concentration of serine and fit to Equation 1 where f is the fractional saturation, [Ser] is the concentration of serine, and *K*_dapp_ is the apparent binding constant for serine.

(Equation 1) $$f=\frac{\left(\left[ser\right]+K_{dapp}\right)-\sqrt{{\left(\left[ser\right]+K_{dapp}\right)}^{2}-4\left[ser\right]}}{2}$$

The addition of serine at the higher concentrations led to precipitation of M391I CBS in the full-length or CBS^Δ516–525^ backgrounds, precluding assessment of the *K*_D*app*(Ser)_ value.

### Stopped-flow spectroscopy

Pre-steady state experiments were performed as described (32), on a TgK stopped-flow spectrophotometer. To characterize the kinetics of serine-dependent aminoacrylate formation, CBS^Δ516–525^ or the K384E/N linker variants (8 μM each in 3 ml of 20 mM HEPES pH 7.4, containing 0.5 mM TCEP and ± 380 μM AdoMet), was rapidly mixed with ten concentrations of serine to obtain final concentrations ranging from 50 to 950 μM (for CBS^Δ516–525^ and K384E CBS^Δ516–525^) or 0.5 to 20 mM (for the K384N CBS^Δ516–525^ variant). The CBS concentration was calculated per 63-kDa monomer and the concentrations are reported prior to mixing in the stopped flow spectrometer.

Data were recorded at 466 nm for 60 s (CBS^Δ516–525^) or 300 s (K384E/N CBS^Δ516–525^) at 20 °C. The kinetic traces were fit to 2 exponentials according to Equation 2. The dependence of the rate constants on serine concentration showed a linear or hyperbolic (Equation 3) behavior for the first and second rate constants, respectively.

(Equation 2) $$A_{466}=A_{o466}-\Delta A_{466_{1}}e^{\left(-k_{obs1}t\right)}-\Delta A_{466_{2}}e^{\left(-k_{obs2}t\right)}$$

(Equation 3) $$A_{466}=\frac{k_{{obs}_{2}}*\left[ser\right]}{K_{d}+\left[ser\right]}$$

To characterize the kinetics of homocysteine-dependent aminoacrylate disappearance, CBS^Δ516–525^ or the K384E/N linker variants (7 μM each in 3 ml of 40 mM HEPES pH 7.4, containing 0.5 mM TCEP and ± 380 μM AdoMet) was preincubated for 15 min at room temperature with 80 μM L-serine to form the aminoacrylate intermediate. The samples were then rapidly mixed with eight concentrations of D,L homocysteine (corresponding to 100, 200, 300, 400, 500, 600, 700, and 800 μM final concentration of the L-isomer, as reported in the figure) and reaction of the aminoacrylate was monitored at 466 nm. Data were fit to the single exponential equation (for CBS^Δ516–525^ ± AdoMet), and double exponential for the linker variants (-AdoMet) according to Equations 2 and 4 respectively.

(Equation 4) $$A_{466}=A_{o466}+\Delta A_{466_{1}}e^{\left(-k_{obs}t\right)}$$

### Structure of K384N-CBS ^Δ411–551^

Initial screens for K384N ^Δ411–551^ were set up in a 96-well sitting drop format, using the commercially available PEG/Ion screen from Hampton Research. Hits were then replicated in a 24-well sitting drop experiment and crystals of K384N^Δ411–551^ were grown at room temperature in a 1:1 mixture of protein (16.6 mg/ml) to well solution (22% PEG 3,350, 6% Tacsimate pH 6.0). Crystals were soaked in a solution of 23% PEG 3.350, 7% Tacsimate pH 6.0, 25% glycerol for 4 h prior to flash cooling in liquid nitrogen.

Diffraction data were collected at LS-CAT (21-ID-D) at Argonne National Laboratory. The data were indexed and integrated using DIALS in Xia2 and scaled in Aimless (43). The structure of K384N ^Δ411–551^ CBS was solved by molecular replacement using a previously solved structure of ΔCBS^Δ411–551^ (PDB code: 1JBQ) using Phaser (44) in the CCP4 program suit. The crystals belonged to the space group P1 (90.07, 89.38, 104.23, 105.51, 106.15, 107.47) with six chains per asymmetric unit. Iterative rounds of model building and refinement were performed with COOT (45) and Phenix (46) or Refmac (47). Ligand restraints were generated in *eLBOW* (48). The geometric quality of the model was assessed in *MolProbity* (49). Data processing and model refinement statistics are reported in Table 5. Models were analyzed and structure figures generated using UCSF Chimera (50).

## Data availability

All data are contained within the manuscript. The coordinates for the K384N CBS^Δ411–551^ (PDB: 8STW) have been deposited in the PDB database.

## Supporting information

This article contains supporting information.

## Conflict of interest

The authors declare that they have no conflicts of interest with the contents of this article.
