# Supplementary material for: Disease-causing cystathionine β-synthase linker mutations impair allosteric regulation
Source: J Biol Chem. 2023 Nov 8;299(12):105449. doi: 10.1016/j.jbc.2023.105449 (PMC10746528; doi:10.1016/j.jbc.2023.105449)
Supplement: Supporting Figures S1 and S2 [file mmc1.docx]

**SUPPLEMENTARY INFORMATION**

**Disease-causing cystathionine b-synthase linker mutations impair allosteric regulation**

Joseph V. Roman, Romila Mascarenhas, Karanfil Ceric, David P. Ballou and Ruma Banerjee*

Department of Biological Chemistry, University of Michigan Medical Center,

Ann Arbor, MI 48109-0600

Corresponding Author: *Address correspondence to: Ruma Banerjee, 4220C MSRB III, 1150 W. Medical Center Dr., University of Michigan, Ann Arbor, MI 48109-0600, Tel: (734)-615-5238; email address: [rbanerje@umich.edu](mailto:rbanerje@umich.edu)

Figure S1

Figure S2

*
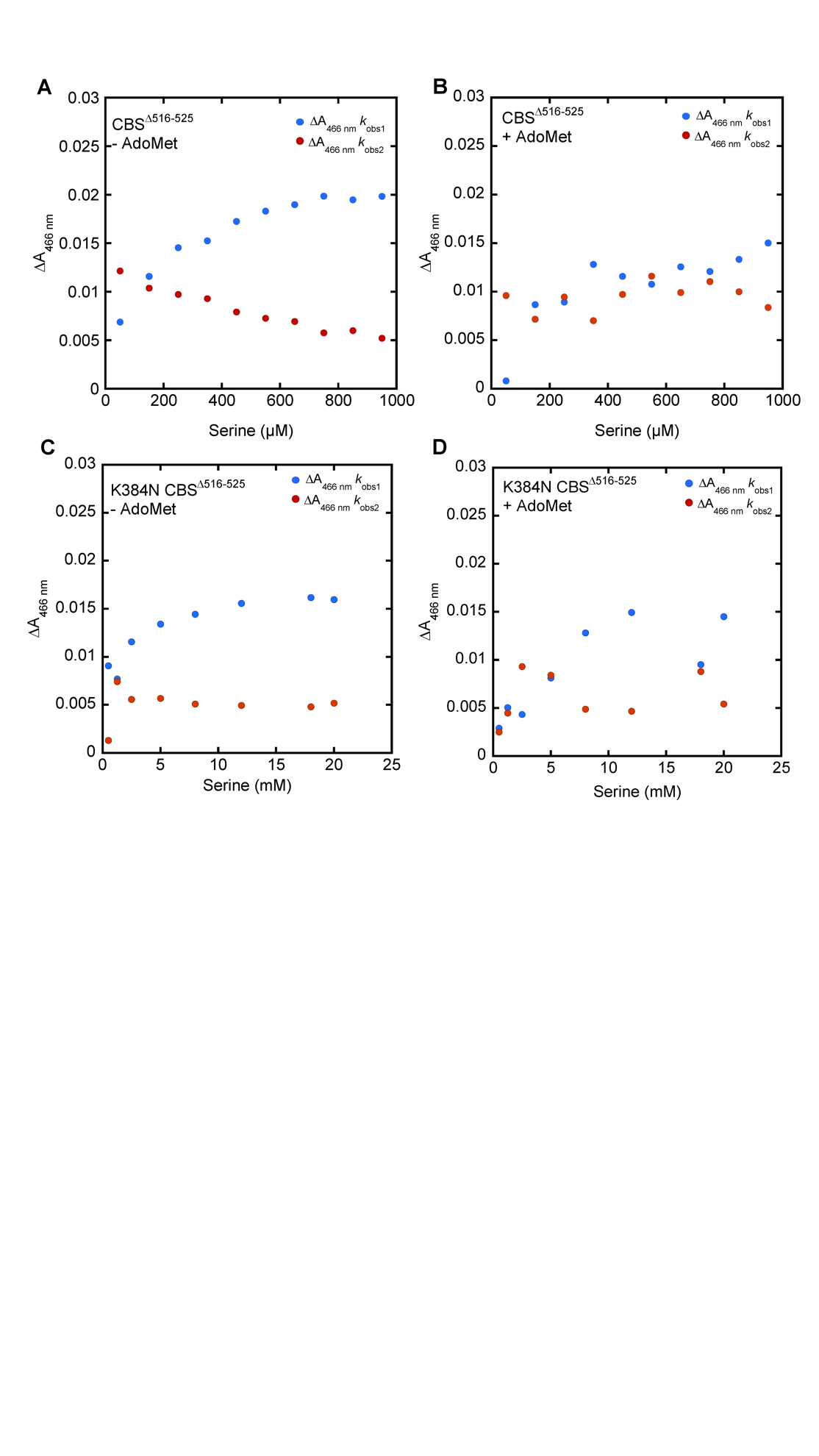
*

**Figure S1. Amplitude changes associated with the reaction of CBS variants with serine. A,B.** Dependence of the amplitude changes associated with *k*_obs1_ and *k*_obs2_ on serine concentration for CBS^Δ516-525^ in the absence (A) or presence (B) of AdoMet. **C, D**. Dependence of ΔA_466 nm_ associated with *k*_obs1_ and *k*_obs2_ on serine concentration for K384N CBS^Δ516-525^ in the absence (C) or presence (D) of AdoMet. The kinetic traces for these reactions are shown in Figs. 4 (for A,B) and 5 (for C,D), respectively. The data are representative of 3 (CBS^Δ516-525^) or 2 (K384N CBS^Δ516-525^) independent experiments each performed with 2 technical replicates.

**
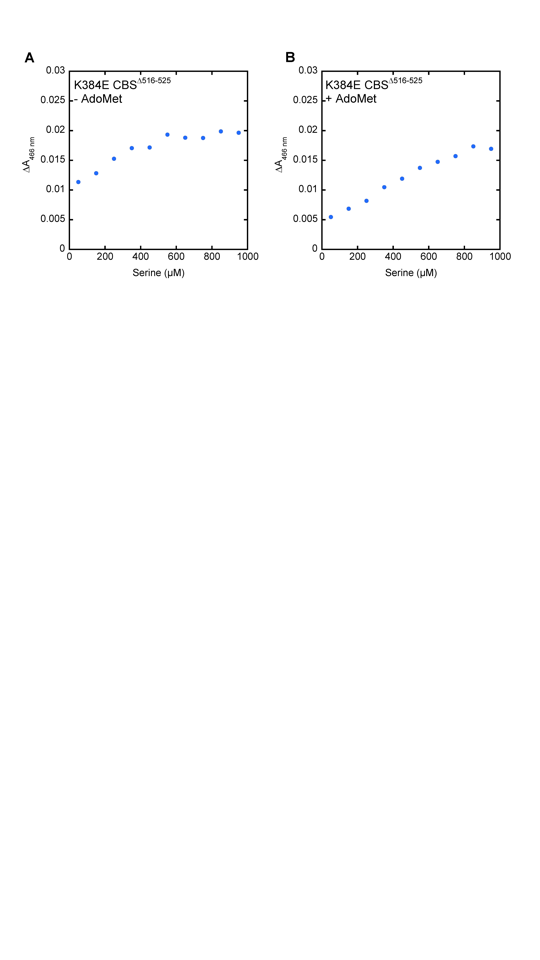
**

**Figure S2. Amplitude changes associated with the reaction of K384N CBS^Δ516-525^ with serine. A,B.** Dependence of ΔA_466 nm_ associated with *k*_obs_ and on serine concentration in the absence (A) or presence (B) of AdoMet. The kinetic traces for these reactions are shown in Fig 6. The data are representative of two independent experiments each performed with 2 technical replicates.
